# Microbiota and anaphylaxis: emerging insights into immune regulation and clinical implications

**DOI:** 10.1097/ACI.0000000000001184

**Published:** 2026-08-03

**Authors:** Martina Ottoni, Antonio Nouvenne, Erminia Ridolo

**Affiliations:** a Allergy, Clinical Immunology and Rheumatology Unit, ASST Carlo Poma, Mantua; b Department of Medicine and Surgery, University of Parma, Parma, Italy

**Keywords:** anaphylaxis, dysbiosis, mast cells, microbiota, short-chain fatty acids

## Abstract

**Purpose of review:**

Anaphylaxis is a life-threatening systemic hypersensitivity reaction with highly variable clinical expression and unpredictable severity. Increasing evidence suggests that host–microbiota interactions may contribute to interindividual variability in allergic sensitization and effector responses. This review summarizes current mechanistic, preclinical, and emerging human data supporting a role for the microbiota in modulating anaphylaxis risk and severity.

**Recent findings:**

Recent experimental studies demonstrate that gut microbial composition and function influence susceptibility to systemic allergic reactions through effects on immune maturation, epithelial barrier integrity, and mast cell biology. Microbiota-derived metabolites, particularly short-chain fatty acids, can directly suppress mast cell activation and degranulation via epigenetic mechanisms. In parallel, preclinical models indicate that dysbiosis exacerbates anaphylactic responses, whereas microbial restoration attenuates disease severity. Emerging translational evidence further suggests that commensal bacteria may directly metabolize food allergens, reducing IgE-binding capacity and effector cell activation. Human studies, although limited, report associations between microbial signatures, allergen-specific IgE levels, and clinical phenotypes, as well as links between microbiota composition and oral immunotherapy outcomes.

**Summary:**

Current evidence supports a biologically plausible role for the microbiota in shaping anaphylaxis susceptibility and severity; however, findings remain largely associative. Future longitudinal and mechanistic studies are needed to establish causality and evaluate the translational potential of microbiota-targeted strategies for the prevention and management of anaphylaxis.

## INTRODUCTION

Anaphylaxis represents the most severe manifestation of systemic hypersensitivity reactions that may result in life-threatening airway, respiratory, and/or cardiovascular compromise [1]. According to the World Allergy Organization (WAO) 2020 and European Academy of Allergy and Clinical Immunology (EAACI) 2021 guidelines, the diagnosis is primarily clinical and relies on the acute onset of mucocutaneous manifestations associated with respiratory compromise, persistent gastrointestinal symptoms, or hypotension with evidence of end-organ hypoperfusion [1,2]. Importantly, following exposure to a known or likely allergen, anaphylaxis can be diagnosed even in the absence of cutaneous manifestations when bronchospasm, laryngeal involvement, or hypotension occurs [1]. Although traditionally viewed as a mast cell-driven disorder, anaphylaxis is now recognized as a complex syndrome involving multiple immune and nonimmune effector pathways that ultimately converge in the release of vasoactive and pro-inflammatory mediators [1,3]. Mechanistically, anaphylaxis can be classified into immunologic, nonimmunologic, and idiopathic forms, highlighting the heterogeneity of the underlying pathogenic mechanisms [3]. Immunologic anaphylaxis includes both Immunoglobulin (Ig)E-mediated and IgE-independent mechanisms, such as IgG-mediated and complement-mediated reactions, whereas nonimmunologic forms result from direct activation of effector cells (mast cells, basophils) or other non-immune-mediated pathways [3–5]. The severity of anaphylaxis is highly variable and remains difficult to predict at the individual level [6]. A previous anaphylactic reaction does not reliably predict the characteristics of subsequent episodes; indeed, even within the same patient, reactions triggered by the same allergen may differ substantially in eliciting dose, onset kinetics, organ involvement, and clinical severity [7]. This heterogeneity reflects not only the dynamic interplay between allergen-related factors (including dose and route of exposure) and recognized cofactors such as physical exercise, nonsteroidal anti-inflammatory drugs (NSAIDs), alcohol consumption, infections, and sleep deprivation but also the biological threshold prevailing at the time of exposure [8]. This threshold is influenced by multiple host-related factors, including intestinal permeability, mast cell and basophil activation status, the systemic inflammatory status, concomitant medications such as angiotensin-converting enzyme (ACE) inhibitors and β-blockers, cardiovascular and respiratory comorbidities, and potentially the composition and metabolic activity of the gut microbiome [5,6,8,9]. Over the past two decades, the microbiota has emerged as a key determinant of interindividual variability in immune responses and, more recently, as a potential contributor to susceptibility to severe allergic reactions and anaphylaxis. The term microbiota refers to the heterogeneous community of microorganisms that colonize a specific body site, including bacteria, archaea, viruses, fungi, and protozoa [10]. Accumulating evidence supports the concept that the microbiota acts as a systemic regulator of immune reactivity through the production of microbial metabolites, modulation of epithelial barrier integrity, and immune imprinting [11,12]. Through these mechanisms, the microbiota influences both mucosal and systemic immune responses, shaping immune tolerance, inflammatory pathways, and susceptibility to allergic diseases [12]. This review summarizes current knowledge and emerging evidence regarding the role of the microbiota in modulating immune reactivity, predisposition to severe allergic reactions, with particular emphasis on the mechanisms through which host–microbiota interactions may influence anaphylaxis susceptibility, clinical expression, and severity. 

**Box 1:**
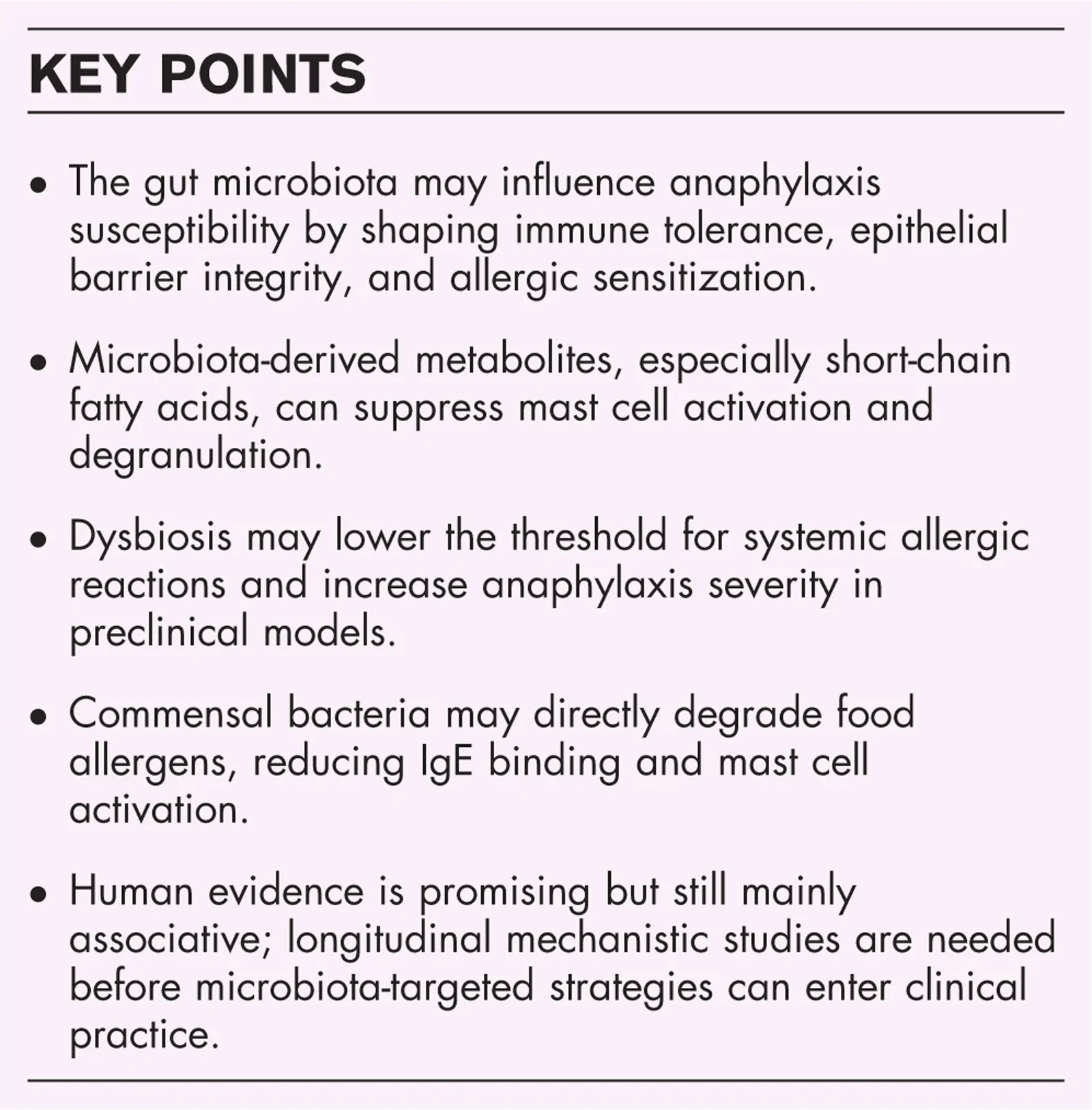
no caption available

## THE MICROBIOTA AS A REGULATOR OF ALLERGIC SENSITIZATION

The development of the gut microbiota during early life is increasingly recognized as a critical determinant of immune maturation and oral tolerance. Several early-life exposures (including cesarean delivery, antibiotic use, reduced or absent breastfeeding, and adherence to a Western diet) have been consistently associated with an increased risk of allergic sensitization and food allergy, supporting the concept that early microbial dysbiosis may contribute to disease pathogenesis [13,14]. Birth mode represents one of the earliest determinants of microbial assembly. Vaginal delivery promotes vertical transmission of maternal anaerobic commensals, including *Bifidobacteria*, whereas cesarean section is associated with delayed microbial colonization, characterized by reduced abundance of beneficial commensals and increased representation of environmental or opportunistic microorganisms [13,15]. Similarly, early-life antibiotic exposure may reduce microbial diversity, deplete communities responsible for producing immunoregulatory metabolites, and induce long-lasting alterations in the complex interactions among the microbiota, intestinal epithelium, and mucosal immune system [13,14]. In contrast, breastfeeding offers a protective effect through the supply of human milk oligosaccharides, secretory IgA, microbial communities, and bioactive metabolites that selectively promote the expansion of *Bifidobacterium* spp. and other taxa associated with physiological immune maturation [15]. A recent study has shown that early colonization by bifidobacterial strains capable of producing aromatic lactates is associated with a reduced risk of food allergen-specific IgE sensitization and subsequent atopic manifestations, highlighting a potential mechanistic role for microbiota-derived metabolites in modulating IgE-mediated immune responses [16]. Conversely, a Western diet, characterized by low fiber intake and high consumption of saturated fats, refined sugars, and ultra-processed foods, may impair microbial metabolic activity, reduce the production of short-chain fatty acids (SCFAs), modify mucus layer homeostasis, and compromise epithelial barrier integrity [17,18].

From an immunological perspective, current evidence suggests that the effects of dysbiosis on food allergy development are mediated through three interconnected pathways: impaired induction of regulatory T cells (Tregs), enhanced type 2 immune responses, and disruption of epithelial barrier integrity. Reduced production of tolerogenic microbial metabolites, including SCFAs and indoles derivatives, can impair Treg induction and promote Th2 polarization, resulting in enhanced production of IL-4, IL-5, and IL-13, IgE class switching, and subsequent mast cell and basophil activation [17–19].

Collectively, these findings support a model in which early-life microbial ecosystems actively shape susceptibility to food allergy through coordinated effects on barrier function, immune regulation, and allergen responsiveness. Importantly, the observation that microbial alterations often precede clinical disease suggests that dysbiosis may represent a permissive factor in the development of IgE-mediated food allergy, providing a strong rationale for microbiota-targeted preventive strategies aimed at restoring immune tolerance during critical developmental windows [13,14,16].

## MICROBIOTA–MAST CELL AXIS: MECHANISTIC LINK TO ANAPHYLAXIS

**FIGURE 1:**
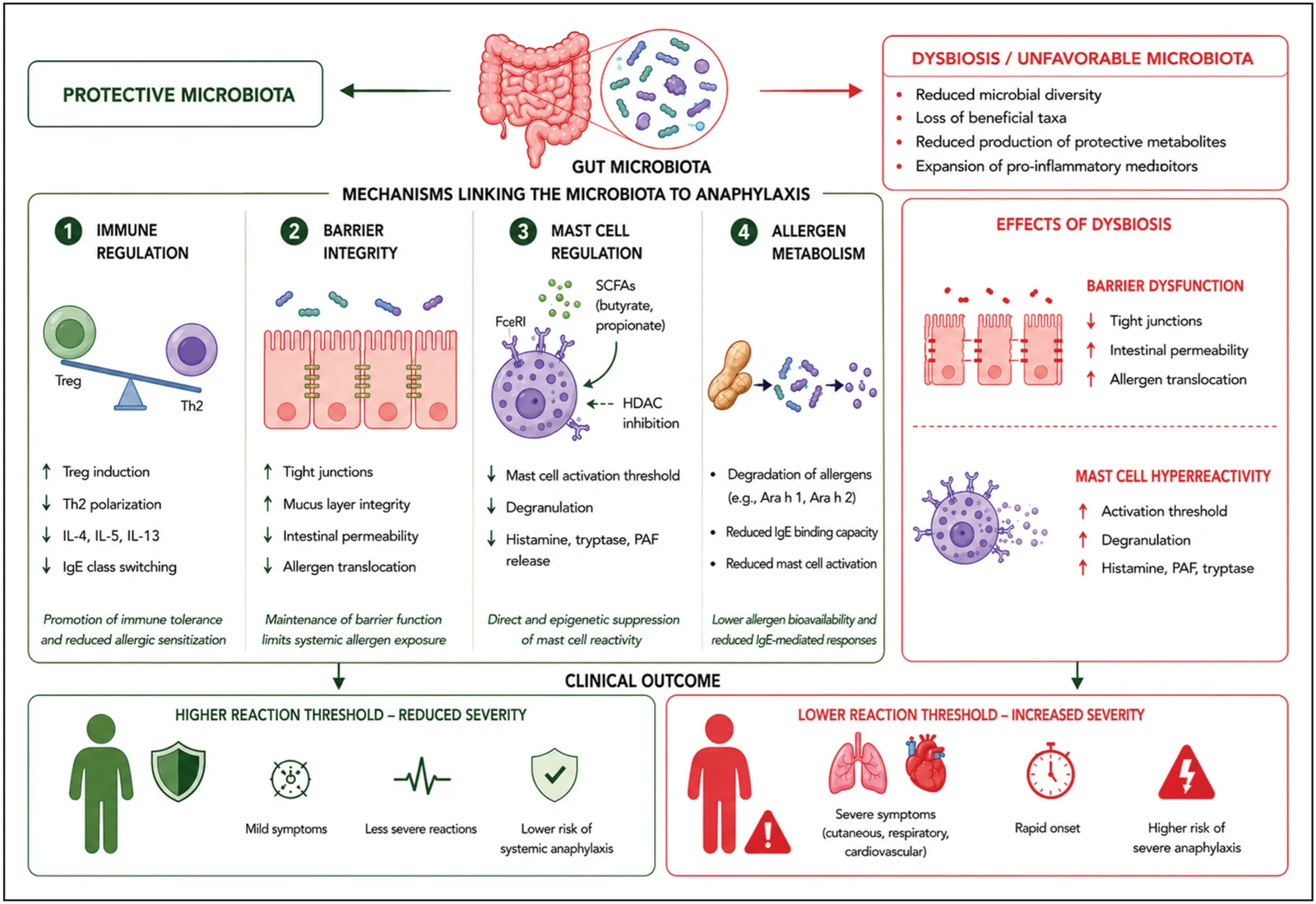
Proposed mechanisms linking the gut microbiota to anaphylaxis. A protective microbiota promotes immune tolerance, preserves epithelial barrier function, restrains mast cell activation, and may reduce allergen bioavailability through direct microbial metabolism. In contrast, dysbiosis is associated with barrier dysfunction, enhanced mast cell reactivity, and a lower threshold for severe anaphylactic responses. Ara h 1 and Ara h 2, major peanut allergens; HDAC, histone deacetylase; IgE, immunoglobulin E; IL, interleukin; PAF, platelet-activating factor; SCFAs, short-chain fatty acids; Th2, type 2 T-helper cell; Treg, regulatory T cell.

Mast cells are the principal effector cells of anaphylaxis, particularly in IgE-mediated reactions. Following allergic sensitization, allergen-specific IgE antibodies bind the high-affinity FcεRI receptor expressed on the mast cell surface. Upon subsequent allergen exposure, cross-linking of the IgE–FcεRI complex triggers intracellular signaling pathways that culminate in mast cell degranulation and the rapid release of preformed and newly synthesized mediators [20]. Histamine and tryptase are among the most widely recognized markers of mast cell activation. Histamine contributes to vasodilation, increased vascular permeability, bronchoconstriction, and cutaneous manifestations, whereas serum tryptase is considered the gold-standard biomarker of systemic mast cell activation [20]. Platelet-activating factor (PAF) represents an additional key mediator, being strongly associated with hypotension, bronchospasm, and severe anaphylactic reactions. Collectively, these mediators account for the rapid onset and potentially life-threatening nature of anaphylaxis [20]. Emerging evidence indicates that mast cell development and responsiveness are not solely determined by host-intrinsic factors but are profoundly influenced by tissue-specific environmental cues, including signals derived from the microbiota. Experimental studies have demonstrated that the absence of a commensal microbiota profoundly alters the mast cell compartment. Germ-free mice exhibit defects in mast cell maturation and intestinal homing, suggesting that microbial colonization is required for the functional development of mucosal mast cell populations [21]. More recent studies have further shown that, under specific genetic and sensitization conditions, germ-free animals display enhanced anaphylactic hypothermia, increased intestinal mast cell accumulation, and elevated levels of mast cell protease-1 (MCPT-1) in both intestinal tissue and serum, supporting a role for the microbiome in determining susceptibility thresholds to anaphylaxis [22]. Although not entirely concordant, these findings collectively indicate that microbiota-mediated regulation of mast cell biology is highly context-dependent and influenced by host genetics, tissue compartment, sensitization protocols, and microbial composition. Recent reviews focusing on the microbiota–mast cell axis in intestinal homeostasis and food allergy have highlighted the coordinated contribution of Toll-like receptor (TLR)-mediated signaling and microbiota-derived metabolites in shaping mast cell function and allergic disease susceptibility [23]. Mast cells express several innate immune receptors, including TLR2 and TLR4, which recognize microbial-derived ligands such as lipoproteins, peptidoglycan, and lipopolysaccharide. Activation of these pathways modulates mast cell maturation, survival, cytokine production, and activation thresholds, thereby influencing downstream allergic responses [23,24]. Among microbial metabolites, SCFAs—primarily acetate, propionate, and butyrate—represent the best-characterized link between diet, the microbiota, and immune regulation [11]. Generated through bacterial fermentation of dietary fibers, SCFAs exert broad effects on both mucosal and systemic immunity by promoting regulatory T-cell differentiation, reinforcing epithelial barrier integrity, and limiting allergic inflammation [11,25^▪^]. Increasing evidence suggests that SCFAs may also directly or indirectly restrain mast cell activation. In particular, butyrate and propionate suppress mast cell activation and degranulation in both murine and human mast cells, reducing histamine release and inhibiting both IgE-dependent and selected IgE-independent activation pathways through histone deacetylase (HDAC) inhibition, which promotes the epigenetic downregulation of key signaling molecules involved in mast cell activation, including *BTK*, *SYK*, and *LAT *[26]. Conversely, antibiotic-induced gut dysbiosis in adult mice reduced immunoregulatory metabolites, including SCFAs, impaired barrier integrity, and increased susceptibility and severity of food allergy, with higher serum histamine and mouse mast cell protease-1 (mMCP-1) after allergen challenge [27]. Collectively, these observations identify the microbiota–mast cell axis as a critical regulator of anaphylaxis susceptibility and severity. Figure 1 summarizes the major mechanisms through which the microbiota may influence mast cell function and anaphylaxis susceptibility.

## MICROBIOTA AND SEVERITY OF ANAPHYLAXIS

Recent evidence suggests that the microbiota influences not only the risk of allergic sensitization but also the severity of anaphylactic reactions.

### Preclinical evidence

In murine models of ovomucoid-induced food allergy, disruption of gut microbial diversity is associated with exacerbated systemic anaphylaxis, increased intestinal mast cell accumulation, and elevated MCPT-1 levels. Adjuvant-free models further demonstrate that both host genetic background and microbiota composition critically shape susceptibility to epicutaneous sensitization. Consistently, germ-free mice exhibit enhanced mast cell activation and more severe anaphylactic responses [22]. Under dysbiotic conditions, exposure to the same allergen may elicit a more severe systemic response through coordinated effects on epithelial barrier integrity, Th2/IgE immunity, and mast cell activation [28]. These findings support the concept that the microbiota contributes to defining the activation set point for anaphylaxis. Moreover, restoration of a protective microbial ecosystem has been shown to attenuate allergic disease severity in experimental settings. Fecal microbiota transplantation in neonatal mice with ovalbumin-induced food allergy reduced allergic manifestations, corrected dysbiosis, and modulated key immunoregulatory pathways [29]. Similarly, recent preclinical studies indicate that microbiota-targeted interventions, including inulin-gel-based oral immunotherapy and fecal microbiota transplantation, can attenuate anaphylaxis severity by remodeling gut microbial communities, reducing anaphylactic symptoms, suppressing Th2/IgE responses, and limiting mast cell-associated mediators such as histamine and mMCP-1 [29,30]. Furthermore, Yakabe *et al. *[31] showed that acarbose-mediated remodeling of microbial carbohydrate metabolism increases gut-derived succinate, which suppresses mast cell activation and attenuates the severity of systemic anaphylactic responses. Collectively, these studies suggest that a protective microbiota may mitigate anaphylaxis severity through effects on both upstream mechanisms of oral tolerance and downstream effector pathways. An innovative area of investigation concerns the direct microbial metabolism of food allergens.

### Allergen metabolism

Traditionally, the microbiota has been viewed as an indirect regulator of allergic responses through its effects on immune development, epithelial barrier function, and the production of immunomodulatory metabolites. However, recent evidence indicates that commensal bacteria can also directly interact with food allergens, modifying their biological availability and allergenic potential. Human oral and intestinal microbial communities contain bacterial species capable of degrading the major peanut allergens Ara h 1 and Ara h 2, thereby limiting the availability of intact allergens for transepithelial passage and systemic immune exposure [32^▪▪^]. Notably, microbial allergen degradation reduces IgE-binding capacity and mast cell activation, attenuating IgE-mediated effector responses and anaphylaxis severity [32^▪▪^,^33^]. Among the identified peanut-degrading taxa, members of the genera *Rothia* and *Staphylococcus* showed marked, strain-dependent degradative activity, highlighting the importance of specific microbial functions rather than taxonomy alone. Consistent with these findings, peanut-allergic individuals with higher abundance of peanut-degrading bacteria, including *Rothia* spp., exhibited higher clinical reactivity thresholds during controlled allergen exposure [32^▪▪^]. Together, these observations suggest that microbial allergen metabolism may contribute to interindividual variability in anaphylaxis severity and represents a promising target for future microbiota-based interventions.

## HUMAN EVIDENCE LINKING THE MICROBIOTA TO ANAPHYLAXIS

Human evidence directly linking the microbiota to anaphylaxis remains limited; however, emerging data from observational studies in allergic individuals support a biologically plausible association. An overview of the principal human studies in this field is provided in Table 1. In the first microbiome study specifically investigating wheat-dependent exercise-induced anaphylaxis (WDEIA), Du *et al.* identified distinct gut microbial signatures in affected patients compared with healthy controls. Notably, the abundance of specific bacterial taxa, including *Bifidobacterium*, *Oscillospira*, and *Leuconostoc*, was significantly associated with total and ω-5 gliadin-specific IgE levels, further linking gut microbial alterations to allergic sensitization and disease-related immunological markers [34]. A subsequent study from the same group demonstrated that individuals with WDEIA adhering to a wheat-free diet exhibited partial restoration of the gut microbial profile, with several bacterial taxa showing inverse correlations with total IgE and wheat-specific, gluten-specific, and gliadin-specific IgE levels [35^▪^]. Together, these findings suggest that microbial alterations may be linked not only to disease susceptibility but also to clinical disease activity. In addition, multicenter longitudinal analyses, conducted in peanut-allergic individuals undergoing oral immunotherapy, have shown that baseline gut microbial composition and microbiota-associated metabolic pathways – particularly those involved in bile acid and amino acid metabolism – are associated with treatment outcomes, suggesting that microbiota-derived metabolites may contribute to the induction and maintenance of long-term allergen tolerance [36]. Emerging evidence has also implicated microbial factors in drug-induced anaphylaxis. In patients experiencing IgE-mediated perioperative anaphylaxis caused by neuromuscular blocking agents, de Chaisemartin *et al. *[37^▪▪^] reported increased levels of circulating microbial DNA during the acute reaction compared with baseline conditions, raising the possibility that barrier dysfunction and microbial-derived signals may contribute to systemic anaphylactic responses. Although preliminary, these observations support the concept that host–microbiota interactions may extend beyond food allergy and influence multiple anaphylactic phenotypes.

**Table 1:** Human studies linking the microbiota to anaphylaxis

Reference	Study type/population	Main microbiota-related findings	Implications for anaphylaxis
Sánchez-Martínez *et al.*, 2026 [32^▪▪^]	Experimental models and translational human data in peanut allergy	Commensal oral and gut bacteria degraded peanut allergens (Ara h 1 and Ara h 2), reducing IgE binding capacity and mast cell activation; higher abundance of peanut-degrading bacteria was associated with higher clinical reaction thresholds during allergen exposure	Identified direct microbial allergen metabolism as a novel mechanism modulating the severity of IgE-mediated anaphylaxis
Du *et al.*, 2020 [34]	Patients with WDEIA	Fecal microbiota composition differed between WDEIA patients and healthy controls. Total IgE levels were negatively correlated with *Bifidobacterium*, whereas ω-5 gliadin-specific IgE was positively associated with *Oscillospira* and negatively associated with *Leuconostoc.*	First evidence linking gut microbial signatures to immunologic markers in a human anaphylaxis phenotype
Du *et al.*, 2026 [35^▪^]	WDEIA patients following a wheat-free diet	A wheat-free diet was associated with partial normalization of gut microbiota structure toward that of healthy controls. At the taxonomic level, *Bacteroides stercoris* was negatively correlated with total IgE, whereas *Catenibacterium* was negatively correlated with total IgE and wheat-specific, gluten-specific, and gliadin-specific IgE levels, suggesting diet-sensitive microbial signatures related to allergic activity.	Suggested that microbiota alterations are dynamic and may reflect disease activity and allergen exposure
Özçam *et al.*, 2025 [36]	Peanut-allergic children undergoing peanut oral immunotherapy (POISED cohort)	Multiomics analysis of fecal samples showed that POIT failure was associated with a distinct bile acid profile, enhanced microbial amino acid utilization, increased protein hydrolysis capacity, and higher abundance of ptpA, a bacterial hydrolase gene capable of cleaving proline-containing tripeptides present in Ara h 2.	Supports a role for microbiota-derived metabolic functions in the development and maintenance of allergen tolerance
de Chaisemartin *et al.*, 2024 [37^▪▪^]	Patients with IgE-mediated perioperative anaphylaxis, mainly NMBAs-related	Plasma 16S rDNA analysis showed higher circulating microbial DNA during acute anaphylaxis than at baseline. Patients also showed a richer circulating microbiota than controls, with differences in taxa including Enterobacteriaceae, Veillonellaceae, *Escherichia-Shigella, Rhodoferax*, and *Lewinella.*	First study investigating the circulating microbiome in human anaphylaxis, suggesting a potential contribution of microbial translocation and systemic microbial signals

16S rDNA, 16S ribosomal DNA; Ara h 1/2, *Arachis hypogaea* allergens 1 and 2; IgE, immunoglobulin E; NMBAs, neuromuscular blocking agents; POISED, Peanut Oral Immunotherapy Study of Early Intervention for Desensitization; POIT, peanut oral immunotherapy; ptpA, proline tripeptide peptidase A; WDEIA, wheat-dependent exercise-induced anaphylaxis.

Despite these advances, important knowledge gaps remain. Human studies are still limited in number, often underpowered, and highly heterogeneous in terms of patient populations, clinical phenotypes, and analytical approaches, with anaphylaxis rarely investigated as a primary endpoint. Although current findings are promising and support a biologically plausible association between the microbiota and anaphylaxis, the available evidence derives predominantly from observational studies and therefore cannot demonstrate causality. Consequently, it remains unclear whether dysbiosis contributes directly to anaphylaxis susceptibility and severity or reflects secondary effects of allergic disease and its management. Prospective longitudinal cohorts and mechanistically informed clinical studies will be required to define causal relationships and assess the translational potential of microbiota-based biomarkers and therapeutic strategies.

## CONCLUSION

Accumulating experimental and translational evidence supports a meaningful role for the microbiota in modulating both the risk and severity of anaphylaxis through multiple interconnected mechanisms, including immune education, epithelial barrier regulation, and direct modulation of mast cell activation. Preclinical studies consistently show that microbial composition and functional capacity influence susceptibility to systemic allergic reactions and shape the magnitude of effector responses. Emerging human data suggest associations between microbial signatures, allergen-specific IgE responses, and clinical phenotypes of food allergy, while recent findings further indicate that microbiota-derived metabolites, as well as direct microbial processing of allergens, may modulate IgE-binding capacity and downstream mast cell activation, thereby influencing anaphylactic outcomes. Nevertheless, current human evidence remains largely observational, heterogeneous, and insufficient to support causal inference or clinical translation. Key unresolved questions include the temporal relationship between dysbiosis and disease onset, mechanistic specificity, and interindividual variability across distinct anaphylaxis triggers. Future studies should prioritize well-powered longitudinal cohorts, standardized multiomics integration, and mechanistically informed interventional designs to delineate causal pathways. Ultimately, the integration of microbial ecology with immunological and clinical frameworks may enable the identification of robust biomarkers and novel therapeutic strategies aimed at modulating the microbiota to prevent or attenuate anaphylaxis.

## Acknowledgements

*None.*

*Author contributions: M.O. and E.R. conceived the review. M.O. and A.N. performed the literature search. M.O. drafted the manuscript and prepared the figures and tables. E.R. and A.N. critically revised the manuscript. All authors contributed to the article and approved the submitted version.*

### Financial support and sponsorship

*None.*

### Conflicts of interest

*There are no conflicts of interest.*

## REFERENCES AND RECOMMENDED READING

Papers of particular interest, published within the annual period of review, have been highlighted as:

▪ of special interest
▪▪ of outstanding interest
